# SHORT CHAIN FATTY ACID CONCENTRATIONS ASSOCIATE WITH PLATELET MITOCHONDRIAL FUNCTION AND MAY MEDIATE SEX-DIFFERENCES IN OLDER ADULTS

**DOI:** 10.1093/geroni/igae098.2301

**Published:** 2024-12-31

**Authors:** Eva Marts Skoglund, Gargi Mahapatra, Anthony Molina

**Affiliations:** University of California Berkeley, Lawndale, California, United States; University of California San Diego, La Jolla, California, United States; University of California San Diego, La Jolla, California, United States

## Abstract

Some studies have reported that mitochondrial function can differ by sex; however, the metabolic pathways that mediate sex differences remain unclear. Our recent studies demonstrate that mitochondrial function, measured in peripheral blood mononuclear cells (PBMCs) and platelets, differ by sex in adults, 55 years of age and older. In a lipidomic analysis of PBMCs and platelets collected from the same participants, we assessed concentrations for 10 short-chain fatty acids (SCFAs), namely, acetate, propionate, isovalerate, valerate, butyrate, isobutyrate, 3-methylvalerate, 4-methylvalerate, 2-methylbutyrate, and caproate. T-tests comparing SCFA concentrations between males and females revealed sex differences in both PBMCs and platelets. Specifically, SCFA concentrations were significantly higher in platelets obtained from males compared to females. Analysis of metabolic pathways involved in the metabolism of these SCFAs revealed that FA biosynthesis, pyruvate metabolism, glycolysis, and gluconeogenesis pathways are significantly affected in both sexes. Further analysis revealed a greater impact of SCFAs in males than females. To test the impact of SCFAs on mitochondrial bioenergetics, we examined correlations between SCFA concentrations and mitochondrial bioenergetic parameters assessed by respirometry. In both PBMCs and platelets, bioenergetic parameters were correlated with acetate, 3-methylvalerate, 4-methylvalerate, and caproate concentrations. In platelets, SCFAs significantly negatively correlated with basal respiration, ATP-linked respiration, and fatty-acid oxidation (FAO). Similar negative correlations were also observed in PBMCs. Our study indicates that sex-dependent differences in mitochondrial bioenergetics are related to sex specific differences in SCFA concentrations. Future studies are needed to determine if differences in SCFA can underlie differences in mitochondrial function between men and women.

